# Panton-Valentine Leukocidin–Positive CC398 MRSA in Urban Clinical Settings, the Netherlands

**DOI:** 10.3201/eid2905.221717

**Published:** 2023-05

**Authors:** Jairo Gooskens, Marja M. Konstantinovski, Margriet E.M. Kraakman, Jayant S. Kalpoe, Nathalie D. van Burgel, Eric C.J. Claas, Thijs Bosch

**Affiliations:** Leiden University Medical Center, Leiden, the Netherlands (J. Gooskens, M.M. Konstantinovski, M.E.M. Kraakman, E.C.J. Claas);; Regional Public Health Laboratory Kennemerland, Haarlem, the Netherlands (J.S. Kalpoe);; Haga Ziekenhuis, The Hague, the Netherlands (N.D. van Burgel);; National Institute for Public Health and the Environment, Bilthoven, the Netherlands (T. Bosch)

**Keywords:** *Staphylococcus aureus*, MRSA and other staphylococci, antimicrobial resistance, Panton-Valentine leukocidin, healthcare-associated infections, the Netherlands, bacteria

## Abstract

We report detection of Panton-Valentine leukocidin–positive clonal complex 398 human-origin methicillin-resistant *Staphylococcus aureus* L2 in the Netherlands. This hypervirulent lineage originated in the Asia-Pacific Region and could become community-acquired in Europe after recurrent travel-related introductions. Genomic surveillance enables early detection to guide control measures and help limit spread of pathogens in urban settings.

*Staphylococcus aureus* is a frequent cause of community- and healthcare-associated infections. Clonal complex 398 (CC398) is of particular concern because it includes common livestock-associated methicillin-resistant *S. aureus* (LA-MRSA) IIa subclades in Europe and human-origin MRSA (HO-MRSA) II-GOI subclades in the Asia-Pacific Region (*1*–*4*). Panton-Valentine leukocidin (PVL)–positive HO-MRSA L2 strains have recently emerged and are expanding geographically, causing skin and soft-tissue infections and occasional invasive disease in humans (*2*,*3*,*5*,*6*).

We report 3 patients in the Netherlands with infections caused by hypervirulent PVL-positive CC398 HO-MRSA L2 strains. The cases were detected as part of a CC398 MRSA surveillance study in a single urban area during 2014–2018 (*7*). One patient had furunculosis skin infection, 1 had a plantar abscess soft-tissue infection, and 1 patient with cystic fibrosis had recurrent bronchitis. One patient had a recent travel history to Vietnam in the Asia-Pacific Region, but none had livestock contact. Phenotypic susceptibility testing of patient samples confirmed methicillin resistance and the MRSA strains were sent to reference laboratories as part of routine molecular surveillance in the Netherlands. Whole-genome sequencing (WGS) and core genome multilocus sequence typing (cgMLST) were performed as part of the CC398 MRSA surveillance study. Detection of 3 PVL-positive CC398 isolates prompted comparative genomic analysis of single-nucleotide polymorphisms (SNPs) outside the scope of the surveillance study. The medical ethical committee of the Leiden University Medical Center approved the study protocol and waived the need for patient consent (approval no. G18.021/SH/sh).

MLST showed sequence types (STs) belonging to CC398, including ST1232 (n = 2), a single-locus variant of ST398, and ST4081 (n = 1), a double-locus variant of ST398. WGS and comparative genomic analysis of SNPs confirmed that all isolates were part of the same L2 lineage within the II-GOI clade (Figure) (*1*–*6*). We performed cgMLST on 1,861 genes, and results showed each patient had a different complex type (CT), CT6700, CT6814, and CT7459, thus ruling out direct transmission events between the patients.

**Figure:**
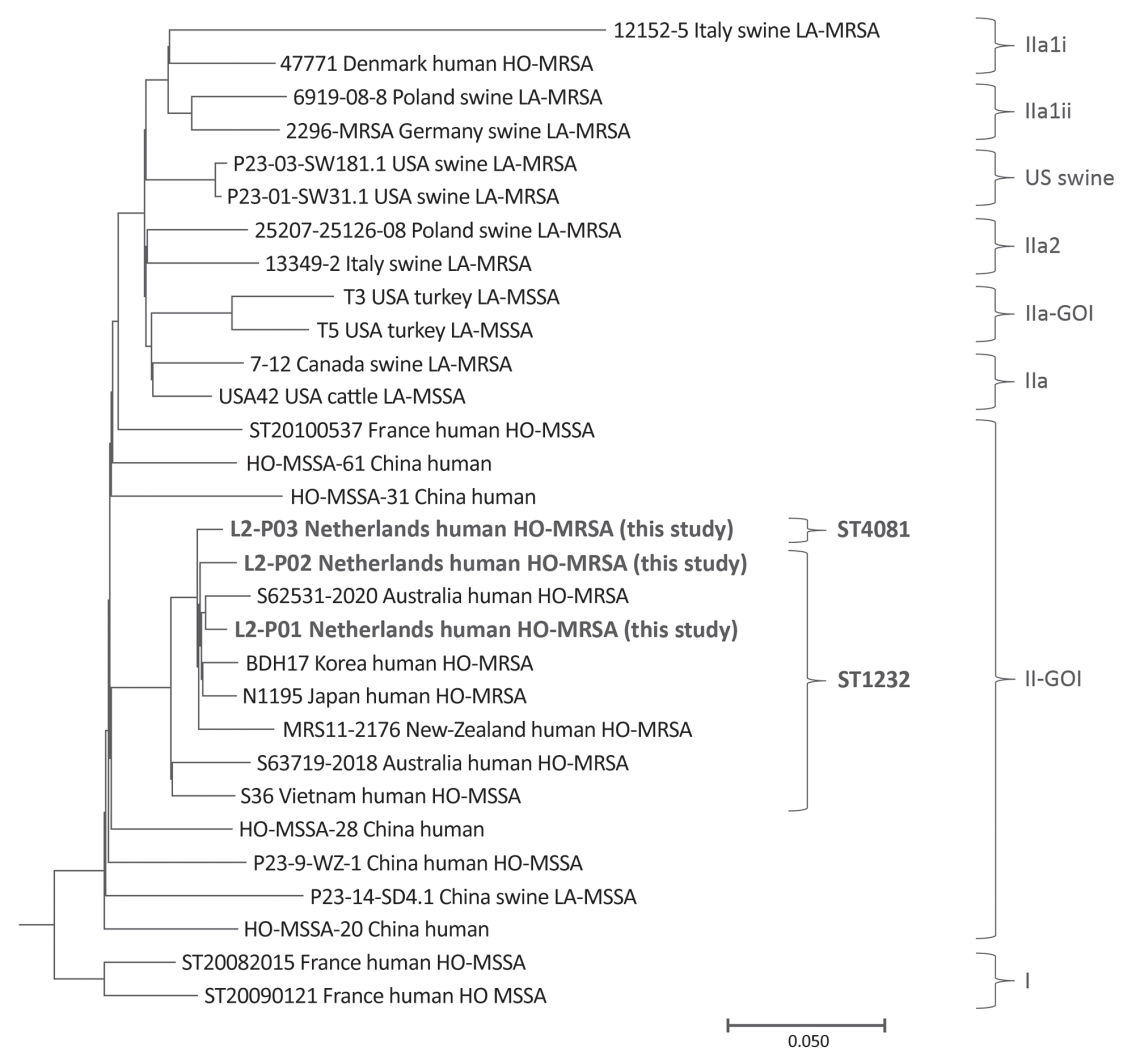
Maximum-parsimony tree of Panton-Valentine leukocidin–positive CC398 MRSA detected in urban clinical settings, the Netherlands. Bold text indicates 3 HO-MRSA strains detected in this study, which we deposited in GenBank (accession nos. SRR21673410 for L2-P01, SRR21624599 for L2-P02, and SRR21673965 for L2-P03); those strains were compared with 27 CC398 *Staphylococcus aureus* strains from GenBank. The tree was constructed by using GenBank accession no. AM990992.1 as a reference strain, and then rooted and grouped into clades, as previously described (*1*). Scale bar indicates nucleotide substitutions per 100 sites based on a concatenated alignment of 3,878 high-quality single-nucleotide polymorphisms. HO-MRSA, human-origin MRSA; LA-MRSA, livestock-associated MRSA; MRSA, methicillin-resistant *Staphylococcus aureus*; MSSA, methicillin-susceptible *S. aureus*; ST, sequence type.

The L2 strains we detected carried resistance genes on the staphylococcal cassette chromosome *mec* element type V(5C2), including *mec*A, which confers β-lactam resistance, and pT181 plasmid integrated *tet*K, which confers tetracycline resistance. In addition, on other mobile genetic elements, the L2 strains carried *erm*(A), conferring macrolide-lincosamide-streptogramin resistance; *ant*(9)-Ia, conferring spectinomycin resistance; *bla*Z, conferring β-lactam resistance; and *tet*38, conferring tetracycline resistance. All 3 strains carried the same virulence markers, including the immune evasion cluster *scn*, *chp*, and *sak*; leukocidin pro-toxin subunits lukF-PV and lukS-PV; *cna*, *ebp*, *ica*, *map*, *sdr*, and *srt*B biofilm formation or microbial surface components recognizing adhesive matrix molecules; immune modulation markers *ads*A, *cap*8, and *sbi*; exoenzymes *aur*, *geh*, *lip*, and *ssp*; and exotoxins or effector delivery components *hla*, *hld*, *hlg*, *esa*, and *esx* (*8*). We deposited the 3 isolates in GenBank (accession nos. SRR21673410, SRR21624599, and SRR21673965) (Figure).

In conclusion, clinicians should be aware of recurrent introductions and evolutionary changes of hypervirulent PVL-positive CC398 HO-MRSA L2 strains in the Netherlands. All 3 patients carrying the detected strains manifested relevant infections, but clinical outcomes were not evaluated in the surveillance study. Additional studies could investigate travel-related transmission routes, disease burden, and clinical outcomes of patients. In addition, future studies could determine if PVL-positive CC398 HO-MRSA strains are becoming established as community-acquired pathogens in urban settings in Europe. We recommend active WGS surveillance for early clinical recognition of PVL-positive CC398 MRSA, which can guide prevention and control measures and limit interhuman transmission and severe clinical outcomes. 
